# How do short sleepers use extra waking hours? A compositional analysis of 24-h time-use patterns among children and adolescents

**DOI:** 10.1186/s12966-020-01004-8

**Published:** 2020-08-14

**Authors:** Aleš Gába, Jan Dygrýn, Nikola Štefelová, Lukáš Rubín, Karel Hron, Lukáš Jakubec, Željko Pedišić

**Affiliations:** 1 Faculty of Physical Culture, Palacký University Olomouc, Olomouc, Czech Republic; 2 Faculty of Science, Palacký University Olomouc, Olomouc, Czech Republic; 3 Faculty of Science, Humanities and Education, Technical University of Liberec, Liberec, Czech Republic; 4 Institute for Health and Sport, Victoria University, Melbourne, Australia

**Keywords:** Sleep duration, Youth, 24-h movement guidelines, Screen time, Time-use epidemiology

## Abstract

**Background:**

To examine compositional associations between short sleep duration and sedentary behavior (SB), light physical activity (LPA) and moderate-to-vigorous physical activity (MVPA) among children and adolescents.

**Methods:**

Multi-day 24-h data on sleep, SB, LPA and MVPA were collected using accelerometers among 343 children (8–13 years old) and 316 adolescents (14–18 years old). Children and adolescents with sleep duration of < 9 and < 8 h, respectively, were classified as short sleepers. Robust compositional regression analysis was used to examine the associations between short sleep duration and the waking-time composition.

**Results:**

Seventy-one percent of children and 75.3% of adolescents were classified as short sleepers. In children, being a short sleeper was associated with higher SB by 95 min/day (*p* < 0.001) and lower MVPA by 16 min/day (*p* = 0.002). Specifically, it was associated with a higher amount of time spent in long sedentary bouts (β_ilr1_ = 0.46, 95% confidence interval [CI] = 0.29 to 0.62) and lower amounts of time spent in sporadic SB (β_ilr1_ = − 0.17, 95% CI = –0.24 to − 0.10), sporadic LPA (β_ilr1_ = − 0.09, 95% CI = –0.14 to − 0.04) and sporadic MVPA (β_ilr1_ = − 0.17, 95% CI = –0.25 to − 0.10, *p* < 0.001 for all), relative to the remaining behaviours. In adolescents, being a short sleeper was associated with a higher amount of time spent in SB by 67 min/day (*p* = 0.001) and lower LPA by 2 min/day (*p* = 0.035). Specifically, it was associated with more time spent in sedentary bouts of 1–9 min (β_ilr1_ = 0.08, 95% CI = 0.02 to 0.14, *p* = 0.007) and 10–29 min (β_ilr1_ = 0.10, 95% CI = 0.02 to 0.18, *p* = 0.015), relative to the remaining behaviours.

**Conclusions:**

Among children and adolescents, short sleep duration seems to be highly prevalent and associated with less healthy waking time. Public health interventions and strategies to tackle the high prevalence of short sleep duration among children and adolescents are warranted.

## Background

Sufficient sleep is essential for physical and mental health [1]. Research has shown that short sleep duration is a risk factor for obesity and poor cardiometabolic health in the pediatric population [2–5]. Additionally, getting enough sleep is associated with enhanced emotional regulation, academic achievement and well-being of children and adolescents [6, 7]. In spite of that, over the last century, a consistent negative secular trend in the sleep duration has been well-documented in these age groups [8]. Due to overuse of electronic devices before sleep onset (e.g., late-night screen time) or absence of household rules and regular sleep-wake routines, insufficient sleep has become a widespread problem [1, 9–11].

School-aged children and adolescents should sleep 9–11 and 8–10 h per 24-h cycle, respectively, to maximize their health [12]. According to the latest estimates more than 40% of children and adolescents from high-income countries do not reach the recommended amount of sleep [11, 13–16]. Among those who do meet the recommendation, sleep constitutes 33–50% of the 24-h cycle. During the remaining time (i.e., waking hours) individuals may engage in a vast range of activities that vary according to their energy expenditure (usually termed as ‘intensity’; e.g. light [LPA] and moderate-to-vigorous physical activity [MVPA]), postural characteristics (e.g., lying back, sitting, standing), and contexts (e.g., at home, in school, in leisure) [17, 18]. Although waking behaviors represent a miscellaneous mixture of behaviors, they can all be classified into two categories: sedentary behavior (SB) and physical activity (PA).

There is a plethora of scientific evidence suggesting that both excessive SB and insufficient PA are associated with similar adverse health outcomes as insufficient sleep [19–21]. For this reason, in recent years, researchers from around the world have shown increased interest in how the amounts of time spent in sleep, SB and PA interact with each other and what is their combined effect on health [22–27]. In 2016, an international effort resulted in the development of the first combined guidelines for sleep, SB and PA in Canada [28], and since that time similar guidelines were issued in several countries [29–33] and by the World Health Organization (WHO) [34]. The main purpose of such guidelines is to take into account co-dependency of sleep, SB and PA and encourage children and adolescents to achieve an optimal time-use balance for health. Specifically, in addition to the abovementioned recommendations for sleep duration, school-aged children and adolescents are encouraged to avoid long periods of uninterrupted sitting, limit screen time to 2 h a day, and accumulate at least 60 min a day of MVPA.

The amounts of time spent asleep and in waking behaviors represent exclusive components of time use, and their sum is always equal to 24 h a day. Thus, any change in the proportion of sleeping time inevitably causes a compensatory change in the proportion of one or more waking behaviors. Such properties make the amounts of time spent in sleep, SB and PA typical examples of compositional data [35]. Novel statistical approaches have recently been introduced to public health research, tailored specifically to compositional properties of 24-h time-use data [24, 25, 36–40]. This coincided with technological developments in wrist-worn accelerometry that enabled obtaining detailed, multi-day 24-h data on sleep, SB and PA, with increased participant compliance and reduced non-wear time [41].

To the best of our knowledge, three previous studies in children and adolescents used 24-h accelerometry to explore how sleep duration in associated with the amounts of time spent in SB and PA [14, 16, 42]. However, their findings were largely inconsistent. Besides, only one of the studies [42] used compositional data analysis (CoDA) to account for the compositional properties of sleep, SB and PA data. A key limitation of this study was a relatively small sample size. Therefore, in this study we used multi-day 24-h accelerometer data and CoDA to examine: (1) differences between short and ‘normal’ sleepers in SB and PA patterns and (2) associations between meeting the sleep recommendation and the amounts of time spent in SB and PA.

## Methods

### Study sample

The study sample included Czech children and adolescents aged 8 to 18 years. Seven primary and four secondary schools from both historical regions of Czechia participated in this cross-sectional investigation. The included schools represent urban and rural schools in which students are engaged in the mandated amount of physical education lessons, that is, 90 min a week. Sports schools and schools for pupils with special educational needs were not included in the study. Participants were recruited via information flyers that were provided to students by the school staff after the school management approved the research. A total of 907 children and adolescents agreed to participate in the study on a voluntary basis. Of these, 248 were excluded because they voluntarily withdrew from the study or became ill (*n* = 45), provided incomplete data or data that could not be assessed due to technical failures (*n* = 146), did not meet the criteria for accelerometer wear time (*n* = 37), or provided incomplete sleep logs (*n* = 20). Hence, the final sample comprised 659 participants (58% girls). Detailed characteristics of the study sample are displayed in the Table 1.

**Table 1:** Characteristics of the study sample

	Total *n* = 659		Children *n* = 343		Adolescents *n* = 316		*p*-value^c^
	Mean^a^	var^b^	Mean^a^	var^b^	Mean^a^	var^b^	
Age (years)	13.9	2.8	11.7	1.6	16.3	1.3	< 0.001
Height (cm)	160.5	14.1	151.6	12.0	170.2	8.7	< 0.001
Weight (kg)	52.9	15.0	43.7	11.4	63.0	11.6	< 0.001
BMI^d^ *z*-score	0.2	1.1	0.2	1.1	0.2	1.0	0.559
**Weight status** (% of *n*)^e^							
Underweight	1.4		2.0		0.6		
‘Normal’ weight	76.1		73.2		79.4		
Overweight	17.0		17.2		16.8		
Obese	5.5		7.6		3.2		
**Sleep**							
% of short sleepers (% of *n*)	73.3		71.4		75.3		
Sleep duration (min/day)^f^	490.2	33.1	520.4	32.3	453.7	37.0	< 0.001
Sleep efficiency (%)^g^	86.5	4.7	85.6	4.5	87.5	4.7	< 0.001
**Waking behaviors**^f^							
SB^h^ (min/day)	675.6	54.0	623.8	53.6	735.1	47.0	< 0.001
LPA^i^ (min/day)	229.0	33.8	241.2	36.2	215.4	34.9	0.001
MVPA^j^ (min/day)	45.2	79.0	54.7	77.9	35.6	81.1	0.021

^a^ Geometric mean for time-use components; arithmetic mean for non-compositional continuous variables; percentages for categorical variables

^b^ The part of total variance related to a given time-use component; standard deviation for other variables

^c^ *p*-value from the *t*-test for independent samples, where the first pivot coordinate was used to represent each time-use variable

^d^ Body mass index

^e^ Categories according to body mass index *z*-score

^f^ The respective time-use composition was adjusted to 24 h before the analysis

^g^ Calculated as the percentage of time spent in sustained inactivity periods divided by the sleep duration

^h^ Sedentary behavior

^i^ Light physical activity

^j^ Moderate-to-vigorous physical activity

### Procedure

Data were collected from 2018 to 2019 during regular school weeks in the spring and autumn seasons. The participants were given accelerometers in the classrooms and were informed briefly on how to wear the device properly. The participants were asked to keep daily logs of their sleep (the wake-up time and the time of sleep onset) and accelerometer non-wear time during the measurement period. Participants were also asked about their age and WHO body mass index *z*-score was calculated from measured height and weight using standard methods.

### Assessment of sleep, sedentary behavior and physical activity

Sleep, SB, and PA were assessed using accelerometers ActiGraph GT9X Link or wGT3X-BT (ActiGraph Corp., Pensacola, FL, USA). Participants were asked to wear the device on their non-dominant wrist for 7 days, 24 h a day. The device was removed for any activity that would involve its submergence in water for a prolonged period (such as bathing or swimming). Accelerometers were set to collect data on three axes at the resolution of 100 Hz using the ActiLife software version 6.13.3 (ActiGraph Corp., Pensacola, FL, USA). The day cycle was determined using “waking up to waking up next day” approach, which means that a full night of sleep is included in a day cycle and that the duration of a day cycle may vary. Accelerometer wear time inclusion criteria were a minimum wearing time of 16 h per day for at least 4 days including at least 1 weekend day [43].

Raw files were processed in the open source R package GGIR, version 1.10–7 through four steps. First, raw signal was auto-calibrated to local gravity [44]. Second, an omnidirectional measure of body acceleration (using Euclidean Norm Minus One [ENMO] metric) and accelerometer’s z-angle were calculated over 5-s epochs. Third, non-wear time and abnormally high acceleration values were detected using the default setting [45]. Last, the data missing due to non-wear time or abnormally high acceleration values (0.7% of accelerometer data) were imputed using the accelerometer data from the same time interval on the remaining days of the week. A comprehensive description of the data processing method is available elsewhere [46].

A default sleep algorithm guided by participant’s sleep logs identified sustained inactivity periods (defined as any period with no change larger than 5° over at least 5 min), sleep onset and waking time [47]. The sleep duration was defined as the difference between the sleep onset and waking time detected by the algorithm. Children (8–13 years old) and adolescents (14–18 years old) with sleep duration of < 9 and < 8 h, respectively, were classified as short sleepers [12]. Sleep efficiency was defined as the percentage of time spent in sustained inactivity periods divided by sleep duration.

To categorize accelerometer data into waking behaviors, we used previously published ENMO thresholds for SB (< 36 m*g*), LPA (36–200 m*g*) and MVPA (≥201 m*g*) [48, 49]. All waking behaviors were further categorized according to their bout lengths. SB bout categories were defined as: 1–9, 10–29 and ≥ 30 min. LPA bout categories were 1–9 and ≥ 10 min, while we considered only one MVPA bout category defined as ≥1 min. The remaining time spent in each waking behavior, that is, in uninterrupted intervals of a given activity of less than 1 min, was considered as “sporadic” SB, LPA and MVPA. When defining thresholds for bout categories, we consulted previous literature [50] and tried to minimize the number of zero values. A time interval was considered to be a sedentary bout, if 90% of its epochs were below the ENMO threshold for SB. PA bouts were considered to be the periods in which 80% of epochs were within the ENMO thresholds for either LPA or MVPA. The weighted averages for each component of the 24-h day cycle were calculated (average of weekdays * 5 + average of weekend days * 2) / 7). These values were then adjusted to sum to 1440 min.

### Statistical analysis

Statistical analysis was conducted in the IBM Statistical Package for the Social Sciences (SPSS) software, version 23 (SPSS Inc., an IBM Company, Chicago, IL, USA) and in the R Statistical Software, version 3.4.2 (R Foundation for Statistical Computing, Vienna, Austria) using the *robCompositions* package [51]. The CoDA approach was applied for analysing the time-use patterns of waking behaviors among short sleepers and ‘normal’ sleepers. For this purpose, two 24-h time-use compositions were considered. The first one was a 4-part composition representing the relative amounts of time spent in sleep, SB, LPA and MVPA. The second one was a 10-part composition that took into account sleep and SB, LPA and MVPA patterns, that is, the abovementioned categories of these behaviors according to the bout length. A Bayesian-multiplicative approach was used to replace a few zeros values in LPA bouts of ≥10 min and MVPA bouts of ≥1 min [52].

We calculated the robust *centre* (i.e., vector of compositional means) for the two 24-h compositions which allows suppress the influence of outlying observations [39]. The dispersion of time-use data was assessed using the *variation matrix*. The 24-h compositions for short and ‘normal’ sleepers were visualized via ternary diagrams, while the relative differences in the composition were visualized using compositional mean bar plots. T-tests for independent samples was used to test the difference between short and ‘normal’ sleepers in each of the time-use components, with the first pivot coordinate (representing the dominance of a given time-use component over the remaining ones) as the dependent variable. The first pivot coordinate includes all relative information about the first part of the time-use composition. To be able to represent each part of the time-use composition using a single pivot coordinate, in every given analysis we rearranged their order.

A set of robust compositional regression analyses were carried out to determine the association between sleep duration (dichotomous variable with categories “short sleepers” and “‘normal’ sleepers”) and the relative amount of time spent in waking behaviors within the 24-h composition [39]. The 24-h composition was mapped into real Euclidean space using isometric log-ratio (*ilr*) coordinates [53]. The outcome variable in the regression analysis was expressed as the first pivot coordinate (i.e., *ilr*_*1*_), which enables interpretation of regression coefficients in terms of dominance of a given compositional part with reference to the remaining parts of 24-h composition. All regression models were adjusted for sex (dichotomous variable; 0 – boys and 1 – girls), body mass index *z*-score (continuous variable), region (dichotomous variable; 0 – Moravia region and 1 – Bohemia region), and season of data collection (dichotomous variable; 0 – spring and 1 – fall). Models were further adjusted for sleep efficiency which could explain variability in waking behaviors related to daytime tiredness [54, 55]. All analyses were conducted separately for children and adolescents. For both age groups, the sample size was sufficient to ensure statistical power of > 99% in the regression model with 14 explanatory variables for alpha error of 0.05 and assuming at least medium effect size (*f*^*2*^ ≥ 0.15) in the population, according to Cohen [56].

## Results

Characteristics of the study sample are shown in Table 1. Children slept on average 67 min/day longer than adolescents (*p* < 0.001), albeit with on average a 1.9% lower efficiency (*p* < 0.001). When compared to adolescents, children spent on average 111 min/day less in SB (*p* < 0.001), 26 min/day more in LPA (*p* = 0.001) and 19 min/day more in MVPA (*p* = 0.021).

Seventy-one percent of children and 75.3% of adolescents were classified as short sleepers (Table 1). Compared with ‘normal’ sleepers, children and adolescents who were classified as short sleepers slept on average 66 min/day and 64 min/day shorter, respectively (*p* < 0.001 for both age categories). Short sleepers had a significantly higher sleep efficiency than ‘normal’ sleepers (*p* < 0.001 for children, *p* = 0.030 for adolescents). Children who were classified as short sleepers fell asleep on average 43 min later and woke up on average 21 min earlier compared with children who were classified as ‘normal’ sleepers. Adolescents who were classified as short sleepers fell asleep on average 54 min later and woke up on average 14 min earlier than their peers classified as ‘normal’ sleepers (Table 2).

**Table 2:** Differences between short and ‘normal’ sleepers in sleep characteristics and waking behaviors

	Children					Adolescents				
	Short sleepers *n* = 245		Normal sleepers *n* = 98		*p*-value^c^	Short sleepers *n* = 238		Normal sleepers *n* = 78		*p*-value^c^
	Mean^a^	var^b^	Mean^a^	var^b^		Mean^a^	var^b^	Mean^a^	var^b^	
Sleep duration (min/day)^d^	502.6	32.0	568.1	29.9	< 0.001	438.0	36.8	501.8	35.0	< 0.001
Sleep efficacy (%)^e^	86.2	4.5	84.1	4.2	< 0.001	87.8	4.7	86.5	4.3	0.030
Sleep onset (hh:mm)^f^	22:16	39	21:33	38		23:29	50	22:35	39	
Wake-up time (hh:mm)^f^	06:40	31	07:01	35		6:52	39	07:06	34	
SB^g^ (min/day)^d^	647.7	50.3	552.5	54.8	< 0.001	749.8	45.2	682.7	48.1	0.001
LPA^h^ (min/day)^d^	236.9	37.1	250.3	42.7	0.167	216.7	35.7	218.7	34.0	0.035
MVPA^i^ (min/day)^d^	52.8	80.6	69.1	72.7	0.002	35.5	82.4	36.7	83.0	0.972
**Bouts of SB**^g^ **(min/day)**^d^										
sporadic	152.4	14.5	172.5	12.2	< 0.001	97.9	15.1	89.1	16.2	0.049
1–9 min	123.4	12.3	121.8	12.3	0.381	107.1	12.1	100.1	12.5	0.005
10–29 min	112.3	14.5	107.3	14.7	0.596	119.5	14.1	113.6	14.1	0.041
≥ 30 min	242.1	26.1	149.2	27.1	< 0.001	419.3	20.7	373.0	24.2	0.350
**Bouts of LPA**^h^ **(min/day)**^d^										
sporadic	145.4	11.5	153.5	11.3	< 0.001	112.8	11.7	106.1	11.4	0.055
1–9 min	81.1	12.8	79.8	13.7	0.419	80.4	11.9	82.8	11.7	0.821
≥ 10 min	16.8	43.1	13.2	54.5	0.042	22.0	43.4	25.3	34.9	0.185
**Bouts of MVPA**^i^ **(min/day)**^d^										
sporadic	36.9	13.8	43.9	14.0	< 0.001	22.0	16.2	23.3	16.6	0.017
≥ 1 min	17.1	40.5	20.9	29.6	0.052	12.7	42.4	11.4	46.1	0.716

^a^ Geometric mean for time-use components; arithmetic mean for other variables

^b^ The part of total variance related to a given time-use component; standard deviation for other variables

^c^ *p*-value from the *t*-test for independent samples, where the first pivot coordinate was used to represent each time-use variable

^d^ The respective time-use composition was adjusted to 24 h before the analysis

^e^ Calculated as the percentage of time spent in sustained inactivity periods divided by the sleep duration

^f^ Time was detected by an algorithm guided by self-reported wake-up and sleep-onset time. Standard deviation is expressed in minutes

^g^ Sedentary behavior

^h^ Light physical activity

^i^ Moderate-to-vigorous physical activity

**Fig. 1:**
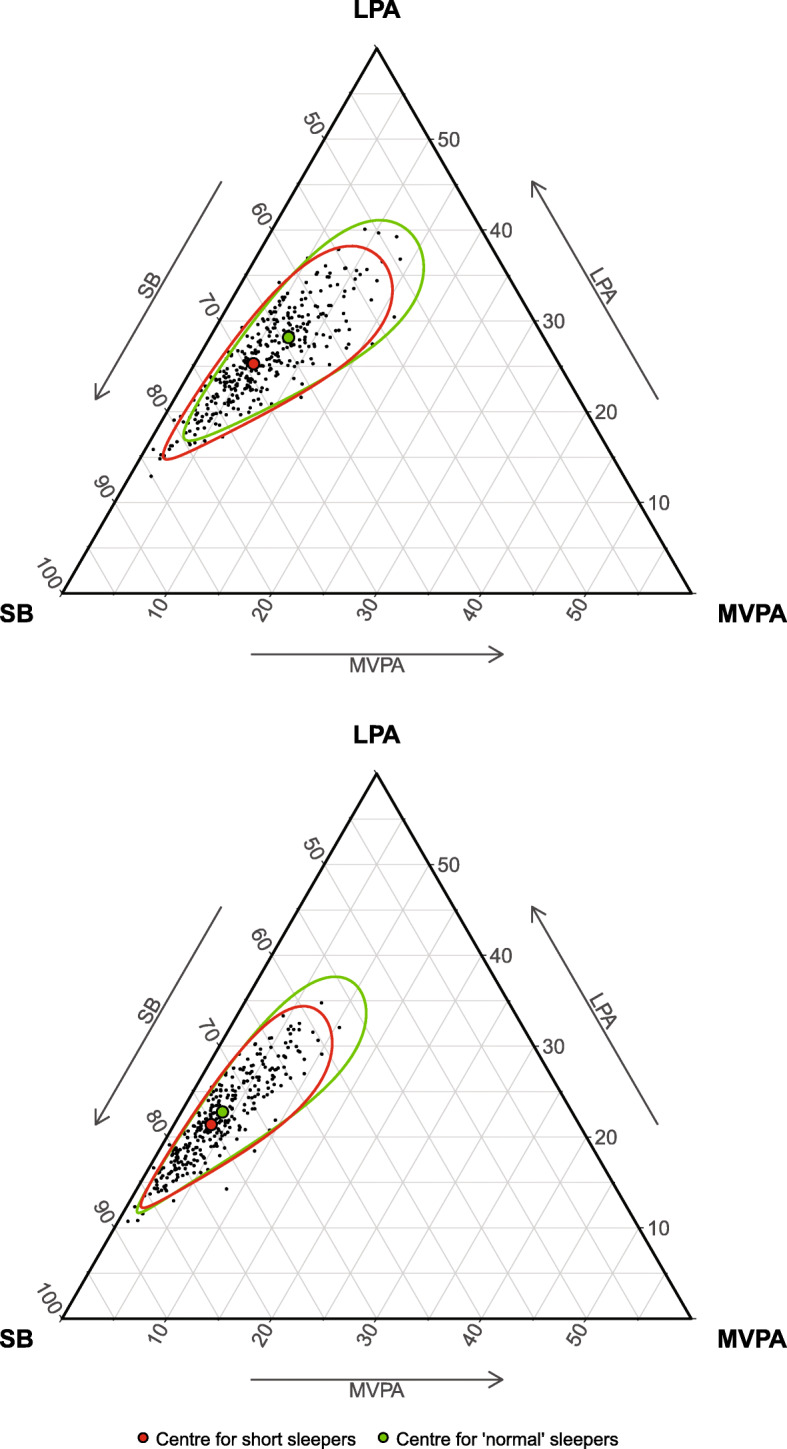
Waking-time composition for short and ‘normal’ sleepers among children (top) and adolescents (bottom). LPA – light physical activity, MVPA – moderate-to-vigorous physical activity, SB – sedentary behavior. Centre represents a vector of compositional means for waking behaviors. The Mahalanobis distance and log-ratio transformation were used to calculate its 95% confidence region

**Fig. 2:**
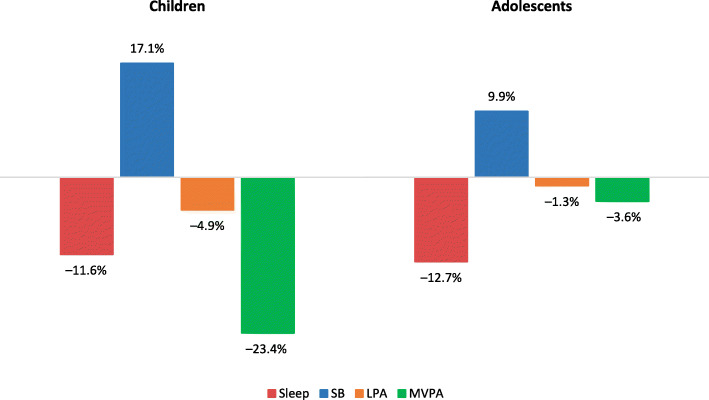
Compositional mean bar plot for a 4-part composition for children and adolescents. LPA – light physical activity, MVPA – moderate-to-vigorous physical activity, SB – sedentary behavior. Bars reflect relative differences in compositional mean of short sleepers relative to compositional mean of ‘normal’ sleepers. The data were centered before the analysis

**Fig. 3:**
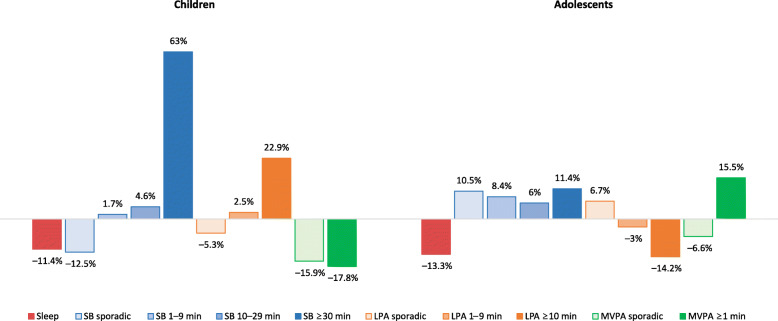
Compositional mean bar plot for a 10-part composition for children and adolescents. LPA – light physical activity, MVPA – moderate-to-vigorous physical activity, SB – sedentary behavior. Bars reflect relative differences in compositional mean of short sleepers relative to compositional mean of ‘normal’ sleepers. The data were centered before the analysis

Among children, short sleepers spent on average 95 min/day (*p* < 0.001) more in SB and 16 min/day (*p* = 0.002) less in MVPA, compared with ‘normal’ sleepers (Table 2). Children who were classified as short sleepers spent on average 93 min/day more in uninterrupted sedentary bouts of ≥30 min (*p* < 0.001) and 4 min/day more in LPA bouts of ≥10 min (*p* = 0.042), compared with those classified as ‘normal’ sleepers. Furthermore, children who were classified as short sleepers spent significantly less time in all sporadic waking behaviors (*p* < 0.001) compared with ‘normal’ sleepers. Furthermore, adolescent short sleepers spent on average 67 min/day more in SB (*p* = 0.001) and 2 min/day less in LPA (*p* = 0.035), compared with ‘normal’ sleepers. Adolescents who were classified as short sleepers spent on average 7 min/day more (*p* = 0.005) in short (i.e., 1–9 min) sedentary bouts and 6 min/day more (*p* = 0.041) in medium (i.e., 10–29 min) sedentary bouts, compared with those classified as ‘normal’ sleepers. Adolescents who were classified as short sleepers spent on average 9 min/day more (*p* = 0.049) in sporadic SB and 1 min/day (*p* = 0.017) less in sporadic MVPA, compared with their peers classified as ‘normal’ sleepers. The differences in 24-h composition between short and ‘normal’ sleepers are further illustrated in Figs. 1, 2 and 3.

Several significant associations between short sleep duration and the components of the two 24-h compositions were found using the compositional regression analysis (Table 3). In children, being a short sleeper was associated with a higher amount of time spent in SB (relative to the remaining behaviors; *p* < 0.001). Being a short sleeper in this age group was also associated with a higher amount of time spent in sedentary bouts of ≥30 min and lower amounts of time spent in sporadic SB, LPA and MVPA (*p* < 0.001 for all), relative to the remaining behaviors. In adolescents, being a short sleeper was associated with a higher amount of time spent in SB (relative to the remaining behaviors; *p* = 0.002). Being a short sleeper in this age group was also associated with higher amounts of time spent in sedentary bouts of 1–9 min (*p* = 0.007) and 10–29 min (*p* = 0.015), relative to the remaining behaviors.

**Table 3:** Results of multivariate robust compositional regression analyses of the associations between sleep duration (explanatory variable) and the amounts of time spent in waking behaviors (outcome variables)

	Children ***n*** = 343			Adolescents ***n*** = 316		
	β_ilr1_^a^	95% CI^b^	***p***-value^c^	β_ilr1_^a^	95% CI^b^	***p***-value^c^
*Model 1*						
SB^d^ (min/day)	0.18	(0.12, 0.24)	< 0.001	0.13	(0.05, 0.21)	0.002
LPA^e^ (min/day)	0.03	(−0.01, 0.06)	0.137	0.04	(−0.01, 0.09)	0.106
MVPA^f^ (min/day)	−0.09	(−0.17, 0.00)	0.053	−0.01	(−0.13, 0.12)	0.914
*Model 2*						
**Bouts of SB**^d^ **(min/day)**						
Sporadic	−0.17	(− 0.24, − 0.10)	< 0.001	0.10	(0.00, 0.19)	0.058
1–9 min	−0.03	(− 0.09, 0.02)	0.208	0.08	(0.02, 0.14)	0.007
10–29 min	0.01	(−0.08, 0.09)	0.868	0.10	(0.02, 0.18)	0.015
≥ 30 min	0.46	(0.29, 0.62)	< 0.001	0.10	(−0.07, 0.27)	0.235
**Bouts of LPA**^e^ **(min/day)**						
Sporadic	−0.09	(−0.14, − 0.04)	< 0.001	0.05	(0.00, 0.10)	0.071
1–9 min	0.02	(−0.04, 0.09)	0.469	−0.03	(−0.08, 0.02)	0.289
≥ 10 min	0.15	(−0.06, 0.37)	0.164	−0.13	(−0.35, 0.08)	0.222
**Bouts of MVPA**^f^ **(min/day)**						
Sporadic	−0.17	(−0.25, − 0.10)	< 0.001	−0.09	(− 0.19, 0.00)	0.062
≥ 1 min	−0.13	(− 0.30, 0.04)	0.145	0.03	(−0.18, 0.24)	0.765

^a^ Non-standardized regression coefficient for the first pivot coordinate. Models were adjusted for sex, body mass index z-score, region, season of data collection, and sleep efficiency

^b^ 95% confidence interval for β

^c^ *p*-value for β

^d^ Sedentary behavior

^e^ Light physical activity

^f^ Moderate-to-vigorous physical activity

## Discussion

We found a very high prevalence of insufficient sleep duration among children and adolescents. Short sleeping was associated with more SB in both age groups. Among children, being a short sleeper was associated with a higher amount of time spent in long, uninterrupted bouts of SB and lower amounts of sporadic SB, LPA, and MVPA. Among adolescents, being a short sleeper was associated with more time spent in sedentary bouts of 1–9 min and 10–29 min.

We found that nearly three quarters of children and adolescents slept too little. The percentage of short sleepers in our study was higher than in a previous large international study in which 59% of participants did not sleep enough [14]. By contrast, the prevalence of short sleepers in our study was lower than among children from Spain [16] in which only 7% of children had sufficient sleep duration. However, these comparisons should be taken with caution, because cut-points for identifying short sleepers and algorithms to detect sleep from accelerometer data differed across studies. In the current study we used a novel algorithm designed to detect nocturnal sleep as a period of sustained inactivity from raw data collected using wrist-worn accelerometers [47], whilst the previous studies used algorithms based on accelerometer counts obtained from waist-worn [14] and wrist-worn devices [16]. It is known that the accelerometer placement site and sleep algorithms may affect estimates of sleep duration obtained from the 24-h accelerometry [57, 58].

We also found that short sleepers fall asleep later in the night than ‘normal’ sleepers. We have no empirical data to explain reasons for the late sleep onset among short sleepers in our sample. However, we can hypothesize it might have been associated with higher late-night screen time. Findings from Hysing and colleagues [59] would support this assumption, as they found in their large population-based study that the vast majority of adolescents use electronic devices before they go to sleep. They also found that the use of electronic devices at bedtime was associated with sleep onset latency of more than 1 h and a reduced sleep duration. In support of these findings, two systematic reviews [10, 60] found that screen time is adversely associated with several sleep outcomes, including sleep duration and sleep onset.

Another reason for children and adolescents to fall asleep late might be that their parents/guardians did not set adequate household rules that would ensure they go to bed on time. Previous studies have found that parents/guardians play a significant role in shaping the sleeping habits of their children [9]. Moreover, adolescents had more delayed sleep onset than children. This finding might be explained by changes in sleep-wake timing that occur during the transition from childhood to adolescence [61]. Our study also revealed that short sleepers woke up earlier than ‘normal’ sleepers. Waking up too early in the morning is considered as a sleep disturbance often caused by poor sleep hygiene or circadian rhythm disorders [62, 63].

As opposed to our results, the only previous study on the association between sleep duration and waking behaviors among children that was based on CoDA [42] found no evidence to suggest that short sleep duration would increase SB or reduce MVPA. It might be that the findings of that study were somewhat compromised by a relatively small sample size and consequently lower statistical power. Our findings on the association between sleep duration and waking behaviors are, however, consistent with some previous studies that were not based on CoDA. For example, the study by Lin and colleagues [14] found that an increase in sleep duration was associated with a significant decrease of SB and increase of both LPA and MVPA during the following day. In contrast to the previous studies and our findings, Sorić and colleagues [64] demonstrated that an extra hour spent in bed during the night was associated with on average 16 min lower MVPA. The inconsistent findings between studies further support the necessity of incorporating appropriate statistical methods for dealing with time-use data, that allow a shift from univariate paradigms to a time-use epidemiology approach and adequately acknowledge compositional properties of time-use data [36]. The use of the same (adequate) statistical methods across studies, would improve the comparability of results.

One of the original findings of the present study is that, among children, short sleep duration is associated with a greater proportion of time spent in long sedentary bouts (i.e., ≥30 min of uninterrupted SB). It is possible that, due to lack of sleep, short sleepers feel too tired to engage in PA and to break their SB more often. Ortega and colleagues [55] found that morning tiredness is associated with lower odds of participating in leisure-time PA and higher odds of excessive screen time, which is in line with our findings. This SB pattern has been associated with increased risk of several adverse health outcomes among children and adolescents, such as adiposity [65] and cardiometabolic markers [66, 67].

The key strengths of the current study include: 1) multi-day, accelerometer-based assessment of time-use behaviors, 2) analysis based on estimates from raw accelerometer data (i.e., ENMO metric), 3) the use of CoDA, and 4) a detailed analysis of SB, LPA and MVPA patterns by categorising these time-use components into different bout lengths.

Our findings need to be interpreted in light of the following limitations. First, accelerometer does not directly assess wakefulness. Thus, our estimates of sleep duration are only an approximation of the true sleep duration. Second, although the sleep algorithm took into account the information participants provided in the sleep logs, the self-reported sleep time window could contain time spent in various bedtime behaviors, such as the time spent trying to fall asleep or night awakenings and attempting to fall back asleep [68]. Third, we calculated time-use variables as an average value from all valid days of accelerometry. Another approach would be to examine the association between sleep and waking behaviors in the context of each 24-h cycle separately. It might be that addressing within-subject variability in sleep duration in the analysis would lead to somewhat different results. Last, cross-sectional nature of this study did not allow us to infer about causality. It may therefore be that less healthy waking behaviours lead to short sleep duration (e.g. a higher level of prolonged SB leads to needing less sleep) or that the associations are bidirectional. This needs to be elucidated in future, longitudinal studies or intervention trials.

## Conclusions

Findings from this study have several important public health implications. The prevalence of short sleep duration among children and adolescents is very high. Our findings suggest that children and adolescents do not spend extra waking hours gained by shorter sleep duration in PA but rather in SB. Public health interventions and strategies to tackle the high prevalence of short sleep duration among children and adolescents are warranted. Future studies should use CoDA to explore how the duration of sleep affects waking-time composition on the following day, as well as how the waking-time composition affects sleep duration in the subsequent night.

## Supplementary information

**Additional file 1.**

## Data Availability

The dataset analyzed during the current study is available in the Figshare repository, 10.6084/m9.figshare.12279806.

## Abbreviations

CoDA: Compositional data analysis
ENMO: Euclidean Norm Minus One
LPA: Light physical activity
MVPA: Moderate-to-vigorous physical activity
PA: Physical activity
SB: Sedentary behavior
WHO: World Health Organization
