# Supplementary material for: How do short sleepers use extra waking hours? A compositional analysis of 24-h time-use patterns among children and adolescents
Source: Int J Behav Nutr Phys Act. 2020 Aug 14;17:104. doi: 10.1186/s12966-020-01004-8 (PMC7427741; doi:10.1186/s12966-020-01004-8)
Supplement: Supplementary file 1 — Additional file 1. [file 12966_2020_1004_MOESM1_ESM.docx]

# Calculation of pivot coordinates

The first pivot coordinate $({ilr}_{1})$ represents a (normalized) logratio with one compositional part in the numerator and the geometric mean of the remaining parts in the denominator. The first pivot coordinate can be interpreted in terms of dominance of a given compositional part with reference to the remaining parts. For example, if $SB$ is a part of interest in a composition consisting of $Sleep, SB, LPA, and MVPA$, the first pivot coordinate ${ilr}_{1}^{\left( SB \right)}$ can be calculated as:

$${ilr}_{1}^{\left( SB \right)}=\sqrt{\frac{3}{4}}\ln\left( \frac{SB}{\sqrt[3]{Sleep\times LPA\times MVPA}} \right)$$

If $SB.sporadic$ is a part of interest in a composition consisting of $Sleep, SB sporadic, SB 1-9 min, SB 10-29 min, SB\geq30 min, LPA sporadic, LPA 1-9 min, LPA\geq10 min, MVPA sporadic, and MVPA\geq1 min$, the first pivot coordinate ${ilr}_{1}^{\left( SB sporadic \right)}$ can be calculated as:

$${ilr}_{1}^{\left( SB sporadic \right)}=\sqrt{\frac{9}{10}}\ln\left( \frac{SB sporadic}{\sqrt[9]{Sleep \times SB 1-9 min \times SB 10-29 min \times SB\geq30 min \times LPA sporadic \times LPA 1-9 min \times LPA\geq10 min \times MVPA sporadic \times MVPA\geq1 min}} \right)$$

The following pivot coordinates were calculated for the purpose of this study:

$${ilr}_{1}^{\left( SB \right)}=\sqrt{\frac{3}{4}}\ln\left( \frac{SB}{\sqrt[3]{Sleep\times LPA\times MVPA}} \right)$$

$${ilr}_{1}^{\left( LPA \right)}=\sqrt{\frac{3}{4}}\ln\left( \frac{LPA}{\sqrt[3]{Sleep\times SB\times MVPA}} \right)$$

$${ilr}_{1}^{\left( MVPA \right)}=\sqrt{\frac{3}{4}}\ln\left( \frac{MVPA}{\sqrt[3]{Sleep\times SB\times LPA}} \right)$$

$${ilr}_{1}^{\left( SB sporadic \right)}=\sqrt{\frac{9}{10}}\ln\left( \frac{SB sporadic}{\sqrt[9]{Sleep \times SB 1-9 min \times SB 10-29 min \times SB\geq30 min \times LPA sporadic \times LPA 1-9 min \times LPA\geq10 min \times MVPA sporadic \times MVPA\geq1 min}} \right)$$

$${ilr}_{1}^{\left( SB 1-9 min \right)}=\sqrt{\frac{9}{10}}\ln\left( \frac{SB 1-9 min}{\sqrt[9]{Sleep \times SB sporadic \times SB 10-29 min \times SB\geq30 min \times LPA sporadic \times LPA 1-9 min \times LPA\geq10 min \times MVPA sporadic \times MVPA\geq1 min}} \right)$$

$${ilr}_{1}^{\left( SB 10-29 min \right)}=\sqrt{\frac{9}{10}}\ln\left( \frac{SB 10-29 min}{\sqrt[9]{Sleep \times SB sporadic \times SB 1-9 min \times SB\geq30 min \times LPA sporadic \times LPA 1-9 min \times LPA\geq10 min \times MVPA sporadic \times MVPA\geq1 min}} \right)$$

$${ilr}_{1}^{\left( SB \geq30 min \right)}=\sqrt{\frac{9}{10}}\ln\left( \frac{SB\geq30 min}{\sqrt[9]{Sleep \times SB sporadic \times SB 1-9 min \times SB 10-29 min \times LPA sporadic \times LPA 1-9 min \times LPA\geq10 min \times MVPA sporadic \times MVPA\geq1 min}} \right)$$

$${ilr}_{1}^{\left( LPA sporadic \right)}=\sqrt{\frac{9}{10}}\ln\left( \frac{LPA sporadic}{\sqrt[9]{Sleep \times SB sporadic \times SB 1-9 min \times SB 10-29 min \times SB\geq30 min \times LPA 1-9 min \times LPA\geq10 min \times MVPA sporadic \times MVPA\geq1 min}} \right)$$

$${ilr}_{1}^{\left( LPA 1-9 min \right)}=\sqrt{\frac{9}{10}}\ln\left( \frac{LPA 1-9 min}{\sqrt[9]{Sleep \times SB sporadic \times SB 1-9 min \times SB 10-29 min \times SB\geq30 min \times LPA sporadic \times LPA\geq10 min \times MVPA sporadic \times MVPA\geq1 min}} \right)$$

$${ilr}_{1}^{\left( LPA\geq10 min \right)}=\sqrt{\frac{9}{10}}\ln\left( \frac{LPA\geq10 min}{\sqrt[9]{Sleep \times SB sporadic \times SB 1-9 min \times SB 10-29 min \times SB\geq30 min \times LPA sporadic \times LPA 1-9 min \times MVPA sporadic \times MVPA\geq1 min}} \right)$$

$${ilr}_{1}^{\left( MVPA sporadic \right)}=\sqrt{\frac{9}{10}}\ln\left( \frac{MVPA sporadic}{\sqrt[9]{Sleep \times SB sporadic \times SB 1-9 min \times SB 10-29 min \times SB\geq30 min \times LPA sporadic \times LPA 1-9 min \times LPA\geq10 min \times MVPA\geq1 min}} \right)$$

$${ilr}_{1}^{\left( MVPA \geq1 min \right)}=\sqrt{\frac{9}{10}}\ln\left( \frac{MVPA \geq1 min}{\sqrt[9]{Sleep \times SB sporadic \times SB 1-9 min \times SB 10-29 min \times SB\geq30 min \times LPA sporadic \times LPA 1-9 min \times LPA\geq10 min \times MVPA sporadic}} \right)$$
